# Macro-environmental factors influencing the preparation of the Ukrainian Olympic team: a comparative PEST analysis of the Tokyo 2020 and Paris 2024 cycles

**DOI:** 10.3389/fspor.2026.1863853

**Published:** 2026-07-02

**Authors:** Olga Kuvaldina, Asta Sarkauskiene, Larysa Taran

**Affiliations:** 1 Department of Sports, Klaipeda University, Klaipeda, Lithuania; 2 Department of Theoretical Foundation of Olympic and Professional Sports, Admiral Makarov National University of Shipbuilding, Mykolaiv, Ukraine; 3 Department of Olympic and Professional Sports, Kharkiv State Academy of Physical Culture, Kharkiv, Ukraine

**Keywords:** crisis, elite sport systems, macro-environment, Olympic sport, PEST analysis, Ukraine

## Abstract

The study examined how macro-environmental factors influenced the preparation of the Ukrainian national Olympic team across two distinct crisis contexts—the Tokyo 2020 and Paris 2024 Olympic cycles. A PEST-based expert survey was applied to assess political, economic, socio-cultural, and technological factors. Two validated questionnaires were used. Twenty-four high-performance athletes who participated in the Olympic Games were involved as experts. The structure of macro-environmental influence differs between the two cycles. In the Tokyo cycle, economic factors had the greatest weight (43%), while political and socio-cultural factors played a more limited role. In contrast, the Paris cycle was characterized by a substantial increase in the importance of political (28%) and socio-cultural (25%) factors, largely related to wartime conditions. Technological factors remained constrained in both cycles. Expert agreement was moderate but statistically significant (Tokyo: *W* = 0.38, *p* < 0.001; Paris: *W* = 0.26, *p* < 0.001). The findings show that macro-environmental influences on Olympic preparation are not stable and shift across crisis contexts. The proposed instrument offers a structured way to assess the direction and relative importance of external pressures in national sport systems.

## 1 Introduction

In recent years, high-performance sport has evolved into a highly complex system. Researchers usually differentiate between macro-level conditions, including policy frameworks, funding structures, and governance, and micro-level environments such as coaching processes, training loads, and athlete development pathways (1–4). These dimensions do not function separately but are shaped by a broader and constantly changing global context. This system has undergone several stages of development, from early international expansion and intensified financial competition to a growing emphasis on innovation, technological advancement, and social impact (5, 6).

Major disruptions, particularly the COVID-19 pandemic, have demonstrated how closely elite sport is embedded in wider socio-political contexts. Under such conditions, sport functions not only as a performance domain but also as a policy instrument and a symbol of national resilience (7). At the same time, crises expose the vulnerability of sport systems and their limited capacity to respond to rapidly changing external conditions (8).

National Olympic Committees are especially sensitive to crises driven by external forces, including pandemics, financial constraints, and armed conflicts (9). These disruptions originate beyond the sport system but require immediate institutional responses, affecting both organizational processes and athlete preparation. Olympic governance should strengthen its ability to anticipate and respond to crisis conditions (10).

In Ukraine, these challenges have overlapped. The COVID-19 pandemic and the full-scale war have disrupted training infrastructure, displaced athletes, and increased pressure on organizational and financial resources (11–15).

Given these crises, relying solely on traditional performance indicators is insufficient. Decision-makers require analytical tools to understand how macro-level pressures impact Olympic preparation and long-term athlete development.

This creates a need for more structured ways to assess how external conditions affect Olympic preparation. One approach for this purpose is the PEST framework, which considers political, economic, socio-cultural, and technological factors. It is widely used in various fields to analyze external environments; however, its application in sport research remains limited, particularly in the context of Olympic sport and national team preparation (16–19).

While some elements such as governance, funding structures, and policy frameworks may be classified as meso-level factors within the Global Sporting Arms Race framework (20), the present study analyzes them through a PEST-based macro-environmental perspective focused on external conditions influencing Olympic preparation.

Existing studies applying PEST analysis in sport are mostly descriptive or combined with broader frameworks such as SWOT, without a clear assessment of the relative influence of individual factors (16–18). The framework itself is also often criticized for remaining at a descriptive level and lacking analytical depth (21, 22), which has been noted in more recent work as well (23).

Research on elite sport systems, in turn, clearly recognizes the importance of macro-environmental conditions, but does not offer many structured ways to assess them in practice (24, 25). This becomes especially problematic in crisis situations, where external pressures intensify and require more precise analysis (9, 10).

However, there is a lack of structured approaches that allow not only identifying but also comparing the relative influence of macro-environmental factors across different crisis contexts in elite sport systems. In the Ukrainian context, no validated instrument currently exists to assess how large-scale disruptions—such as pandemics or war—affect Olympic preparation.

The present study applies a PEST-based framework to assess the relative influence of political, economic, socio-cultural, and technological factors on the preparation of the Ukrainian national Olympic team across the Tokyo 2020 and Paris 2024 Olympic cycles using an expert-based approach.

## 2 Materials and methods

### 2.1 Study design

The study was based on an expert survey and examined macro-environmental conditions affecting the Ukrainian Olympic team under major external shocks. The PEST framework was used to classify macro-environmental factors into political, economic, socio-cultural, and technological categories.

The analysis covered two Olympic cycles: Tokyo 2020 and Paris 2024. These periods developed under clearly different external conditions. Preparation for the Tokyo Games took place during the COVID-19 pandemic, which disrupted qualification events, training processes, and international mobility. In contrast, the Paris cycle unfolded during the full-scale war in Ukraine, affecting infrastructure, logistics, institutional stability, and everyday training conditions.

A retrospective expert assessment was used to evaluate macro-environmental influences across these two Olympic cycles.

### 2.2 Instrument

Two questionnaires were used in the study. The first contained 27 items and referred to the Olympic cycle of the Games of the XXXII Olympiad in Tokyo (2020). The second consisted of 28 items and corresponded to the cycle associated with the Games of the XXXIII Olympiad in Paris (2024).

The items were organized according to the PEST framework and represented political, economic, socio-cultural, and technological factors. Experts evaluated the influence of each factor separately using a scale ranging from −5 to +5, where negative values indicated an adverse influence and positive values indicated a favorable influence, and 0 indicated a neutral position. Experts also assigned weight coefficients to the four PEST domains (political, economic, socio-cultural, and technological) to indicate their relative importance.

Both questionnaires also included open-ended questions for additional comments.

The instruments had previously undergone content validation involving seven experts from four countries (Germany, Lithuania, Poland, and Ukraine). The validation procedure included two rounds of expert evaluation combined with cognitive interviews. All items demonstrated acceptable content validity coefficients (CVC ≥ 0.80) (38).

### 2.3 Participants

The expert sample included 24 high-performance athletes who had participated in the Olympic Games. Of these, 12 athletes participated in the Games of the XXXII Olympiad in Tokyo (2020), including 2 men (16.7%) and 10 women (83.3%). The athletes represented different Olympic sports, including athletics, weightlifting, shooting, and artistic swimming. The Paris 2024 group also included 12 athletes: 5 men (41.7%) and 7 women (58.3%), representing athletics, weightlifting, shooting, diving, and artistic swimming. The participants had between 10 and 20 years of professional sports experience.

### 2.4 Data collection

Data was collected using Google Forms. The questionnaires were distributed electronically to the selected respondents. Participation in the study was voluntary, and all participants provided informed consent prior to completing the survey. Responses were collected anonymously, and the data were used exclusively for research purposes.

The open-ended questions allowed respondents to comment on key factors, advantages, limitations, and possible improvements in each Olympic cycle. These responses were later grouped by meaning and used to support the interpretation of the results.

### 2.5 Data analysis

The data collected was processed using SPSS Statistics. Descriptive statistics were used to summarize expert evaluations.

For each factor, the mean value (M) was calculated based on expert scores.

Kendall's coefficient of concordance (W) was calculated to assess the level of agreement among experts in each questionnaire using the formula (Equation 1):

$$W=\frac{12\sum {\left(R_{j}-R\right)}^{2}}{m^{2}\left(n^{3}-n\right)}$$ (1)

where $R_{j}$ is the sum of ranks for each factor, *R* is the mean of rank sums, *m* is the number of experts, and *n* is the number of factors.

The statistical significance of the obtained coefficients was evaluated using the chi-square (*χ*²) test (Equation 2):

$$\chi^{2}\;=m(n-\;1)W$$ (2)

To determine the relative contribution of each PEST domain, experts assigned proportional coefficients to political, economic, socio-cultural, and technological factors. The mean values for each domain were calculated across all experts and converted into percentages so that the total across the four domains equaled 100% within each Olympic cycle.

## 3 Results

### 3.1 Political factors

The expert evaluation of political factors across the Tokyo 2020 and Paris 2024 Olympic cycles is presented in Table 1.

**Table 1:** Comparison of political factors across the Tokyo 2020 and Paris 2024 Olympic cycles.

Factor code score (M)	Factor description	Mean
Tokyo 2020 cycle—15%		
P1-20	Political situation in Ukraine in 2020 (government changes and administrative instability)	0.50
P2-20	Effectiveness of state policy supporting athletes	1.17
P3-20	Implementation of key national strategic documents in sport policy	0.50
P4-20	International political environment (diplomatic relations, sanctions, IOC decisions)	0.67
P5-20	COVID-19-related political decisions affecting sport (quarantine restrictions, facility closures, event cancellations)	−3.17
P6-20	Postponement of the Games of the XXXII Olympiad in Tokyo and related regulatory adjustments	−3.00
P7-20	Ukraine's diplomatic actions in protecting athletes’ rights and ensuring participation in the Games of the XXXII Olympiad	1.33
Paris 2024 cycle—28%		
P1-24	Impact of hostilities and martial law in Ukraine (2022–2024) on the training process and preparation conditions of the national Olympic team	−4.17
P2-24	State support programs for the preparation of the national team of Ukraine in Games of the XXXІII Olympiad	−0.33
P3-24	War-related relocation of athletes and training bases and its effect on preparation quality	−2.00
P4-24	International political environment in Olympic sport (IOC decisions, participation regulations, boycott of Russian and Belarusian athletes, international solidarity)	−2.00
P5-24	Political pressure and discrimination affecting Ukrainian athletes during participation in the Games of the XXXIII Olympiad in Paris	−1.83
P6-24	Diplomatic support and protection of the interests of the national team during the preparation period (2022–2024)	0.33
P7-24	International cooperation within the Olympic movement (NOC, IOC, federations) in organizing training camps abroad	0.50

In the Tokyo 2020 cycle, political factors accounted for 15% of the total influence, representing one of the lowest contributions among all factor groups.

The main negative effects were pandemic-related political decisions, in particular quarantine restrictions, closure of training facilities, and the postponement of the Games of the XXXII Olympiad in Tokyo, which received the lowest evaluations within this domain.

Positive contributions were mainly related to diplomatic support and international cooperation, while state policy measures had a moderate effect. The general political situation in the country, the implementation of strategic documents, and the international political environment remained close to neutral, reflecting relatively stable background conditions rather than a direct influence.

In the Paris 2024 cycle, the importance of political factors increased to 28%, making them one of the leading domains of the macro-environment. The strongest negative impact was associated with hostilities and the introduction of martial law.

Additional constraints were linked to the relocation of athletes and training bases, as well as to the international political context and instances of political pressure.

Diplomatic support and international cooperation within the Olympic movement remained the main positive components, although their contribution was limited in comparison to the overall scale of negative effects. State support programs were assessed as close to neutral. Overall, political factors became substantially more influential during the Paris cycle under wartime conditions.

### 3.2 Economic factors

Table 2 presents the expert evaluation of economic factors across the Tokyo 2020 and Paris 2024 Olympic cycles.

**Table 2:** Comparison of economic factors across the Tokyo 2020 and Paris 2024 Olympic cycles.

Factor code score (M)	Factor description	Mean
Tokyo 2020 cycle—43%		
E1-20	Level of state funding during the Olympic cycle (2016–2020) for ensuring athlete preparation for the Games of the XXXII Olympiad in Tokyo	0.67
E2-20	Individual state financial support and payments to athletes	0.50
E3-20	Reduction (sequester) of the state budget and funding constraints during the COVID-19 pandemic	−1.33
E4-20	Financial provision of modern sports equipment, gear, and training infrastructure	0.67
E5-20	COVID-19-related quarantine restrictions limiting participation in commercial competitions	−0.67
E6-20	Financial and organizational support from international sports organizations (IOC, Olympic Solidarity, European sport bodies)	0.83
E7-20	Expenditures on travel, training camps, and logistical support of the national team	1.00
Paris 2024 cycle—31%		
E1-24	Level of state funding during the Olympic cycle (2021–2024) for ensuring athlete preparation for the Games of the XXXIII Olympiad in Paris	0.50
E2-24	Individual state financial support and payments to athletes	1.50
E3-24	Impact of the war in Ukraine on the ability to attract financial support from private businesses and commercial sponsors	−1.33
E4-24	Financial provision of modern sports equipment, gear, and training infrastructure during preparation for the Games of the XXXIII Olympiad in Paris	−0.33
E5-24	Impact of martial law and restrictions on travel abroad on participation in professional international competitions involving remuneration, contracts, or sponsorship	−1.67
E6-24	Financial and organizational support from international sports organizations (IOC, Olympic Solidarity, European federations) during 2022–2024	−1.16
E7-24	Wartime funding and its effect on the costs of travel, accommodation, and logistics during Olympic preparation	0.50

In the Tokyo 2020 cycle, economic factors accounted for 43% of the total influence, representing the largest contribution among all factor groups.

The most negative effect was the reduction of the state budget and funding constraints during the COVID-19 pandemic.

The highest positive values were observed for expenditures related to training camps, travel, and logistical support. State funding, individual financial support for athletes, provision of equipment and infrastructure, and support from international sports organizations were also positive, but their values were lower. Restrictions on participation in commercial competitions had a negative effect, although it was less pronounced.

In the Paris 2024 cycle, the share of economic factors decreased to 31%, and their structure became less balanced. The strongest negative impact was associated with restrictions on participation in international competitions under martial law.

Close to this were limitations in attracting private funding and sponsorship, as well as reduced support from international sports organizations. These factors formed the main negative cluster within the economic domain.

The highest positive evaluation was individual financial support for athletes. In contrast, state funding, logistical support, and the provision of equipment and infrastructure showed comparatively weaker effects and did not stand out within the overall pattern. Economic factors remained important across both Olympic cycles, although their relative influence decreased during the Paris cycle.

### 3.3 Socio-cultural factors

The expert evaluation of socio-cultural factors across the Tokyo 2020 and Paris 2024 Olympic cycles is presented in Table 3.

**Table 3:** Comparison of socio-cultural factors across the Tokyo 2020 and Paris 2024 Olympic cycles.

Factor code score (M)	Factor description	Mean
Tokyo 2020 cycle—15%		
S1-20	Social isolation and limited interaction with family, media, and the public during 2020–2021	−1.17
S2-20	Public and fan support (including direct and online communication, public recognition)	−0.17
S3-20	Athlete intrinsic motivation, self-discipline, and stress resilience during the COVID-19 period	−3.00
S4-20	COVID-19-related quarantine restrictions (competition cancellations, mobility limitations, remote training)	−2.17
S5-20	Reduction in interpersonal interaction and team cohesion	−1.33
S6-20	Informational and media support (national media, social networks, sports journalism)	−0.17
Paris 2024 cycle—25%		
S1-24	Psychological state of athletes under wartime conditions (stress, anxiety, uncertainty) and its impact on performance	−1.67
S2-24	Public and fan support during athletes’ participation in the Games of the XXXIII Olympiad in Paris	2.17
S3-24	Moral and motivational factors related to war (sense of national responsibility, representing the country)	0.33
S4-24	War-related displacement of athletes and its effect on social adaptation, communication, and preparation quality	−2.00
S5-24	Limited communication with family during the preparation period and its impact on athletes’ psychological state	−1.83
S6-24	Media support of the national team (social networks, journalism) during preparation and participation	0.67
S7-24	Discrimination against Ukrainian athletes related to the war during preparation and participation	−1.17

In the Tokyo 2020 cycle, socio-cultural factors accounted for 15% of the total influence and did not play a leading role within the overall structure. A clear feature of this period is that all socio-cultural factors were evaluated negatively, indicating a consistently adverse effect across the group.

The strongest negative influence was associated with reduced intrinsic motivation, self-discipline, and stress resilience among athletes during the COVID-19 period. Quarantine restrictions, including cancelled competitions and limited mobility, had a similar effect and further disrupted preparation. Other factors, such as social isolation, reduced interaction, and weaker team cohesion, followed the same pattern but were less pronounced. Public and media support remained limited and did not offset the overall negative trend.

In the Paris 2024 cycle, the share of socio-cultural factors increased to 25%, indicating a more important role compared to the previous period. The most negative influence was related to the displacement of athletes and its impact on the training process.

Similar patterns were observed in relation to limited communication with family and the general psychological strain associated with wartime conditions.

At the same time, the highest positive evaluation was public and fan support, which became a significant source of psychological reinforcement. Media support and motivational factors related to representing the country also showed positive effects, although to a lesser extent. Overall, socio-cultural factors became more influential during the Paris cycle due to the broader psychological and social impact of wartime conditions.

### 3.4 Technological factors

The expert evaluation of technological factors across the Tokyo 2020 and Paris 2024 Olympic cycles is presented in Table 4.

**Table 4:** Comparison of technological factors across the Tokyo 2020 and Paris 2024 Olympic cycles.

Factor code score (M)	Factor description	Mean
Tokyo 2020 cycle—27%		
T1-20	Access to modern training technologies	−1.33
T2-20	Availability of performance monitoring, functional testing, and training load analysis systems	−2.67
T3-20	Technical equipment of training facilities (e.g., video analysis systems, sensor platforms, load monitoring devices)	−1.83
T4-20	Use of advanced research and analytical equipment in athlete preparation	−2.83
T5-20	Access to sports medicine, rehabilitation services, and recovery support	−3.33
T6-20	Use of digital technologies in training (virtual training, online communication, digital monitoring)	−2.50
T7-20	International educational and knowledge exchange initiatives supporting coaching staff during Olympic preparation	−1.17
Paris 2024 cycle—16%		
T1-24	Access to modern training technologies	−1.67
T2-24	Availability of performance monitoring, functional testing, and training load analysis systems	−2.50
T3-24	Technical equipment of training facilities (e.g., video analysis systems, sensor platforms, load monitoring devices)	−1.67
T4-24	Use of advanced research and analytical equipment in athlete preparation	−1.67
T5-24	Access to sports medicine, recovery procedures, and rehabilitation support	−1.17
T6-24	Use of digital technologies in training (virtual training, online communication, digital monitoring)	−1.17
T7-24	International educational and knowledge exchange initiatives supporting coaching staff during Olympic preparation	0

In the Tokyo 2020 cycle, technological factors accounted for 27% of the total influence and formed one of the key domains affecting the preparation process.

All indicators within this group were evaluated negatively, indicating a consistently adverse impact of technological conditions. The strongest constraints were related to limited access to sports medicine and recovery services, as well as insufficient use of advanced research and analytical equipment. Close to this were restricted access to performance monitoring systems and the reduced application of digital technologies in training.

Other technological components followed the same pattern, including access to modern training technologies, the technical equipment of training facilities, and opportunities for international knowledge exchange. Although their values differed, the overall direction remained unchanged.

In the Paris 2024 cycle, the share of technological factors decreased to 16%, and their overall impact became less pronounced.

Negative effects were observed across most indicators, particularly in access to performance monitoring systems and modern training technologies under wartime conditions.

Limitations in technical equipment and the use of analytical tools also remained evident, although their intensity was lower compared to the previous cycle.

At the same time, the differences between factors became smaller. Most of them showed similar levels of influence, without clear contrasts. International educational and knowledge exchange initiatives were close to neutral and did not have a noticeable effect. Overall, technological factors remained constrained across both Olympic cycles despite differences in crisis conditions.

**Figure 1:**
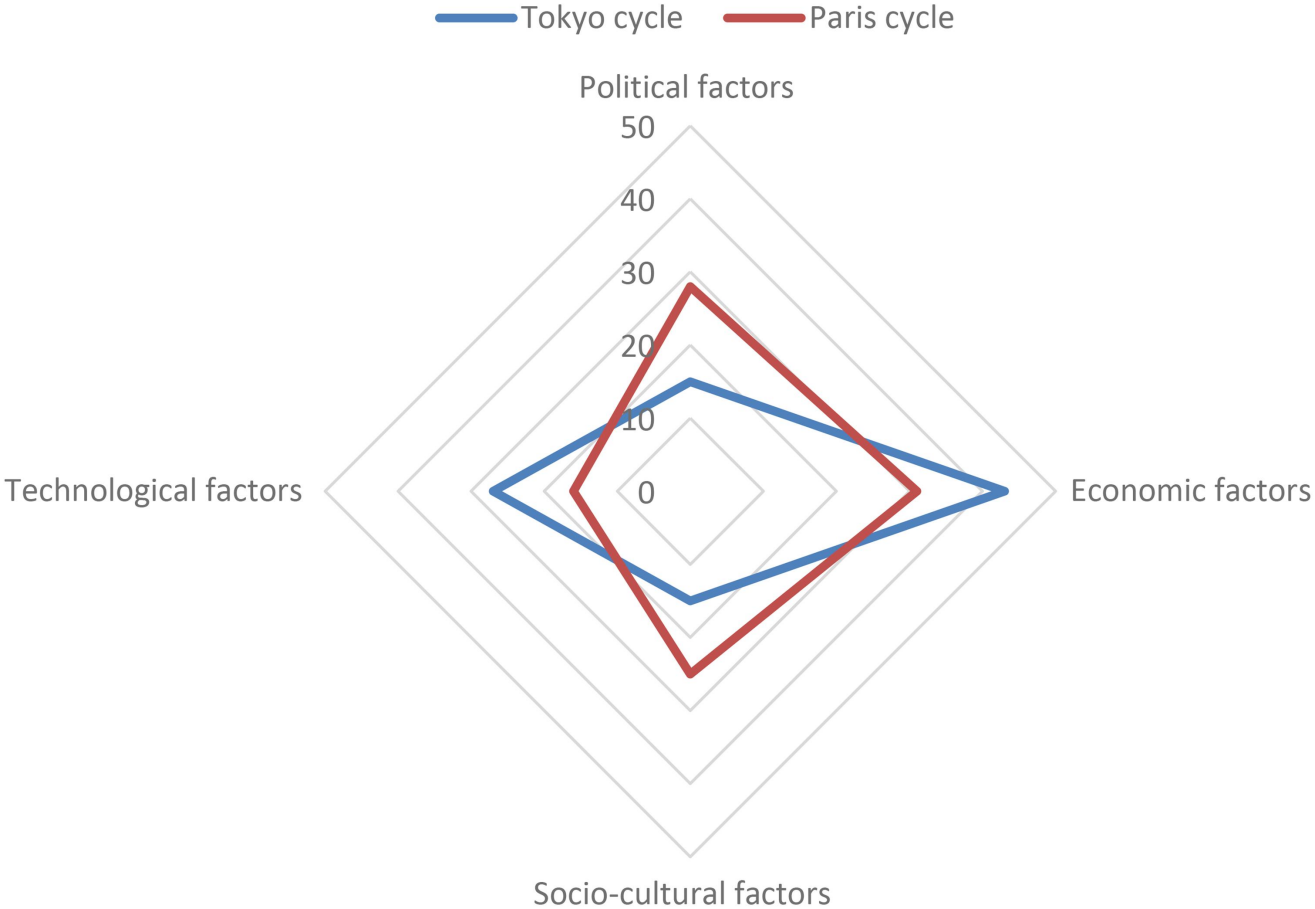
Comparison of PEST factor weights across Olympic cycles (Tokyo 2020 and Paris 2024).

The distribution of PEST factors across the two Olympic cycles is shown in Figure 1.

### 3.5 Expert agreement

For the Tokyo 2020 cycle, Kendall's coefficient of concordance indicated a moderate level of agreement among experts (*W* = 0.38). The calculated chi-square value was *χ*² = 108.7 (df = 26, *p* < 0.001), which confirms that the agreement was statistically significant.

For the Paris 2024 cycle, the level of agreement was lower (*W* = 0.26), indicating greater variability in expert evaluations. At the same time, the result remained statistically significant (*χ*² = 77.2, df = 27, *p* < 0.001).

### 3.6 Additional insights from expert comments

#### 3.6.1 Tokyo 2020 Olympic cycle

Negative factors were mainly associated with the postponement of the Games and the pandemic, which disrupted the preparation process.

In some cases, respondents also mentioned time zone differences, competition shortly after arrival, and limited training conditions.

Positive aspects include motivation, team readiness, and the role of coaches. One response also refers to stable state support as a factor that helped maintain preparation.

Suggested improvements focus on training camps in the host country, financial support, and access to medical, rehabilitation, and psychological services. Some answers also mention better communication and cooperation with decision-makers.

#### 3.6.2 Paris 2024 Olympic cycle

Responses in this cycle are more consistent and mostly focused on the impact of war.

Negative factors relate to reduced resources, limited infrastructure, unstable training conditions, and constant psychological pressure. In several responses, war is directly identified as the main constraint.

Positive aspects are linked to motivation, psychological resilience, and training abroad. Some respondents also mention increased media attention and the importance of communicating the situation in Ukraine.

Suggested improvements focus on financial support, sport psychology, and access to proper training conditions and technologies. One response directly refers to the need to end the war.

Overall, the qualitative responses are consistent with the PEST results. Economic and technological aspects appeared more indirectly, mainly through references to resources, infrastructure, and training conditions.

## 4 Discussion

The results indicate that the influence of the macro-environment on the preparation of the Ukrainian national Olympic team is dynamic rather than stable across Olympic cycles. It changes both in strength and in structure. The comparison between the Tokyo and Paris cycles shows a clear difference: during the pandemic, the situation was constrained but still manageable, whereas wartime conditions created a more disruptive and less predictable environment. This is reflected in the stronger role of political and socio-cultural factors.

The moderate Kendall's W values may reflect differences in how athletes from various Olympic sports perceived external factors affecting preparation. This was particularly evident during the Paris cycle, where wartime conditions influenced sports and athletes differently. Therefore, the findings should be interpreted as reflecting overall tendencies within the Ukrainian Olympic system rather than fully generalizable patterns.

Political factors become more decisive in the Paris cycle. Their influence extends beyond national policy and increasingly depends on the international context, including decisions of sport governing bodies and access to competitions. The importance of institutional and political conditions for elite sport systems has been widely discussed in the literature (4, 5, 24). This is also evident in the Ukrainian context, where the structure of the sport system and its governance arrangements shape how external pressures are absorbed and managed (26). In crisis situations, particularly during war, this dependence becomes more direct, as external decisions begin to define the basic conditions of preparation (27, 28). This is consistent with broader research on global sport governance, where international organizations regulate access to resources, participation, and competition environments (29). As a result, elite sport systems become more externally dependent, especially in contexts such as Ukraine, where institutional capacity to buffer external shocks remains limited.

Economic factors appear more inert. Their relative importance remains high in both cycles, yet their influence does not shift as sharply as political conditions. Financial mechanisms in national sport systems tend to adapt slowly and do not react instantly to external shocks. This pattern is consistent with the structure of sport financing in Ukraine. In 2024, 41% of total sport funding was covered by the state, while 59% was provided at regional and local levels. At the same time, programme expenditures were concentrated in sport schools (33%), high-performance sport (30%), and infrastructure (33%) (26, 30). Such a distribution leaves limited room for rapid financial reallocation under crisis conditions. In addition, external support becomes critical. After 2022, targeted assistance from the International Olympic Committee, Olympic Solidarity, and other international structures helped to offset internal constraints. This support included funding, training opportunities abroad, and access to competitions (13, 31–34). These measures helped maintain a basic level of system stability despite ongoing disruption.

The socio-cultural dimension depends on the type of crisis. During the pandemic, it was mostly linked to restrictions, reduced interaction, and limited support networks. In the wartime context, the situation becomes more complex. Alongside negative effects, new sources of motivation appear. Public attention, international support, and the symbolic value of representing the country in crisis conditions shape how athletes perceive participation (35). The international response to the war—including sanctions and political positioning within the Olympic movement—also contributes to a specific socio-psychological environment (15, 36). In this context, resilience tends to strengthen, although it coexists with significant psychological pressure (14, 37). Under these conditions, socio-cultural factors become more directly embedded in the conditions of preparation. Increased psychological pressure, uncertainty, and the need to adapt to disrupted living and training environments make these factors more influential in shaping how athletes continue their preparation. This helps explain their increased relative importance in the Paris cycle.

Technological factors remain constrained across both Olympic cycles. Limited access to modern training technologies, medical support, and analytical tools persist regardless of external conditions. This suggests that technological capacity is not only shaped by short-term disruptions but is rooted in more structural characteristics of the sport system. Evidence from recent research supports this interpretation. In a Delphi study of Ukrainian Olympic sport under wartime conditions, the provision of modern equipment and training infrastructure was identified among the most urgent needs, ranking fifth among priority areas (11, 13). This indicates that technological limitations cannot be explained by wartime disruption alone. Rather, they reflect a more systemic gap in resource provision and management. Even under conditions of adaptation, these constraints are not fully overcome, pointing to their persistent and structural nature (29).

In this study, macro-environmental factors were assessed in terms of their direction and relative magnitude based on expert evaluation using a validated instrument, allowing a more differentiated understanding of how external conditions shape athlete preparation under crisis conditions. The findings indicate that macro-environmental influences are not stable across crises, as their relative importance and overall configuration vary depending on the context, suggesting that such frameworks should be applied as dynamic analytical tools rather than static classifications of external factors.

In practical terms, this means that under conditions similar to the Tokyo cycle, greater attention should be given to maintaining technological and medical support systems, which appear as persistent constraints. In contrast, under wartime conditions, priority shifts towards managing socio-psychological pressures and ensuring stable access to international training and competition environments.

The results reflect the Ukrainian context and cannot be directly transferred to other countries, where sport systems and external conditions differ. At the same time, the approach used in this study can be applied by other national Olympic committees and sport federations to assess external pressures, identify priority areas, and support planning and resource allocation under unstable conditions.

Limitations. The results are based on expert assessments and therefore reflect perceived rather than directly measured effects. The retrospective design may have introduced recall bias, particularly in relation to the Tokyo 2020 cycle. In addition, the findings reflect the perspectives of athletes only and may differ from the views of coaches, administrators, or other stakeholders within the sport system. The sample size was limited, and the level of agreement between experts was moderate. These aspects should be considered when interpreting the results.

## 5 Conclusions

This study examined macro-environmental factors influencing the preparation of the Ukrainian national Olympic team across the Tokyo 2020 and Paris 2024 Olympic cycles under crisis conditions. The results show that the structure of macro-environmental influence changes across crisis contexts, with economic factors dominating during the pandemic and political and socio-cultural factors becoming more prominent under wartime conditions.

These findings indicate that external conditions do not simply intensify but reconfigure the system of influences affecting Olympic preparation, shifting priorities between different domains. This highlights that macro-environmental factors should be treated as an active and dynamic component of the sport system rather than as a stable background.

The study also demonstrates that combining the PEST framework with expert-based quantitative assessment makes it possible to capture both the direction and relative importance of these influences, providing a clearer basis for analyzing Olympic preparation under conditions of crisis.

## Data Availability

The datasets generated during this study are available from the corresponding author on reasonable request.

## References

[1] Gowthorp L. High-Performance sport. In: Girginov V Sherry E, editors. High-Performance Sport. London: Routledge (2024). 10.4324/9780367766924-RESS75-1

[2] Power MJ Widdop P Parnell D Carr J Millar SR. Football and politics: the politics of football. Managing Sport and Leisure. (2020) 25(1–2):1–5. 10.1080/23750472.2020.1723437

[3] De Bosscher V Shibil S Westerbeek H Van Bottenburg M. Successful Elite Sport Policies : An International Comparison of the Sports Policy Factors Leading to International Sporting Success (SPLISS 2.0) in 15 Nations. Aachen: Meyer & Meyer Sport (2015). p. 400.

[4] Jacobs S De Bosscher V Venter R Patatas JM Scheerder J. Contextual factors influencing the South African elite sporting system: an ‘open system’ approach. International Journal of Sport Policy and Politics. (2021) 13:699–714. 10.1080/19406940.2021.1951327

[5] Van der Roest JW De Bosscher V Shibli S. High-performance sport systems: current developments and future challenges. In: Shilbury D, editor A Research Agenda for Sport Management. Cheltenham: Edward Elgar Publishing (2022). p. 149–66.

[6] Belcastro F. A game of politics? International sport organisations and the role of sport in international politics. Int Spect (Rome). (2023) 58:107–22. 10.1080/03932729.2023.2205789

[7] Elcombe TL. Sport in times of turmoil: political uses of sport in global crises. Glob Soc. (2022) 36:538–61. 10.1080/13600826.2021.1973382

[8] Ratten V da Silva Braga VL da Encarnação Marques CS. Sport entrepreneurship and value co-creation in times of crisis: the COVID-19 pandemic. J Bus Res. (2021) 133:265–74. 10.1016/j.jbusres.2021.05.001

[9] Preuß H Schallhorn C Schütte N. Olympic Sport Organisations in Times of Crisis and Change. Baden-Baden: Academia – ein Verlag in der Nomos Verlagsgesellschaft (2022).

[10] Holger P Christiana S Norbert S. Olympic Sport Organisations in Times of Crisis and Change : Guide for Strategic Management and Good Governance. Baden-Baden: Academia (2022). p. 246.

[11] Kuvaldina O Agostinis-Sobrinho C. Rebuilding dreams: prioritising the needs of Ukraine’s Olympic sports amidst war. Br J Sports Med. (2024) 58:816–7. 10.1136/bjsports-2024-108507 38702183

[12] Driukov O Driukov V Kuvaldina O Biryuk S Kravchuk L. Formulating strategic directions for developing the national sports federation in modern socio-economic conditions. Phys Educ Theory Methodol. (2024) 24:290–7. 10.17309/tmfv.2024.2.14

[13] Kuvaldina O Sarkauskiene A Rybak O Taran L Derkach V Biryuk S. Top 10 needs of Ukraine’s Olympic sports in hostile conditions: a delphi study. BMJ Open Sport Exerc Med. (2024) 10:e001653. 10.1136/bmjsem-2023-001653 38410410 PMC10895238

[14] Purdy LG Kohe GZ Paulauskas R. Professional sports work in times of geopolitical crises: experiences in men’s basketball in Ukraine. Manag Sport Leis. (2023) 28:344–59. 10.1080/23750472.2021.1908842

[15] Kobierecka A Kobierecki MM. Enforced ostracism? Analysis of the international sports Organizations’ reactions to the 2022 Russian invasion of Ukraine. Int J Hist Sport. (2023) 40:1321–45. 10.1080/09523367.2024.2306860

[16] Jedel J Antonowicz P. Evaluation and development prospects of the sports and recreation market in Poland in 2000–2030. PEST analysis from a legal, economic, socio-cultural and technological perspective. Balt J Health Phys Act. (2018) 10:202–12. 10.29359/BJHPA.10.4.19

[17] Wang L Zhao F Zhang G. Analysis on the impact of large-scale sports events on regional economy based on SWOT-PEST model. J Math. (2022) 2022:12. 10.1155/2022/7769128

[18] Zhou J. Strategic analysis of Shenzhen’s sports city development: an exploration based on SWOT and PEST frameworks. J Res Soc Sci Humanit. (2024) 3:53–60. 10.56397/JRSSH.2024.12.07

[19] Dong H Liu Z Kong K Li T Ma Q. Evaluation of input-output efficiency of sports industry based on SWOT-PEST model. J Math. (2021) 2021:1–11. 10.1155/2021/6294745

[20] De Bosscher V. The Global Sporting Arms Race : An International Comparative Study on Sport Policy Factors Leading to International Sporting Success. Meyer & Meyer (2008). p. 173. Aachen: Roundhouse [distributor].

[21] Burt G Wright G Bradfield R Cairns G van der Heijden K. The role of scenario planning in exploring the environment in view of the limitations of PEST and its derivatives. Int Stud Manag Organ. (2006) 36:50–76. 10.2753/IMO0020-8825360303

[22] Berisha Qehaja A Kutllovci E Shiroka Pula J. Strategic management tools and techniques usage: a qualitative review. Acta Univ Agric Silvic Mendel Brun. (2017) 65:585–600. 10.11118/actaun201765020585

[23] Anderson P. The pestel framework and its variants for analysing the strategic environment: Evolution, limitations and adoption in energy policy research (2025).

[24] Ramos R De Bosscher V Pankowiak A Valleser CW. Contexts shaping the development and success of elite sport systems: a scoping review. Sport Management Review. (2023) 26:649–76. 10.1080/14413523.2023.2171276

[25] Gómez-Rodríguez J Seguí-Urbaneja J Teixeira MC Cabello-Manrique D. How countries compete for success in elite sport: a systematic review. Soc Sci. (2024) 13:31. 10.3390/socsci13010031

[26] Rauch RL Kropyvnytska T. Sport system and policy in Ukraine. Int J Sport Policy Politics. (2025):1–18. 10.1080/19406940.2025.2599140

[27] Lee JW. A wartime olympics?: geopolitical tensions, international ambition, and IOC’s vision at Paris 2024. French Politics. (2025) 23:327–45. 10.1057/s41253-025-00293-4

[28] Grix J James M. The politicisation of sport and the principle of political neutrality: a contradiction in terms? Int Sports Law J. (2024) 24:68–77. 10.1007/s40318-024-00273-w

[29] Bayle E. Governance, Regulation and Management of Global Sport Organisations. London: Routledge (2024).

[30] Ministry of Youth and Sports of Ukraine. FINAL REPORT for 2021-2025 on the results of the implementation of the State Targeted Social Program for the Development of Physical Culture and Sports for the period until 2025 (2025).

[31] International Olympic Committee (IOC). Initial USD 200,000 released to support Ukrainian Olympic community (2022). Available online at: https://olympics.com/ioc/news/initial-usd-200-000-released-to-support-ukrainian-olympic-community (Accessed May 31, 2026).

[32] World Athletics. Ukrainian athletes benefit from fund to attend World Athletics Championships. Available online at: https://worldathletics.org/news/press-releases/ukrainian-athletes-benefit-fund-world-championships (Accessed May 31, 2026).

[33] Support for Ukraine: Sport clubs and federations. Available online at: https://www.sportanddev.org/en/article/news/support-ukraine-sport-clubs-and-federations (Accessed May 31, 2026).

[34] IOC. IOC continues to provide widespread support for Ukrainian athletes ahead of Paris 2024 and Milano Cortina 2026 (2023). Available online at: https://www.olympics.com/ioc/news/ioc-continues-to-provide-widespread-support-for-ukrainian-athletes (Accessed May 31, 2026).

[35] Black DR. Sports mega-events and changing world order. Int J. (2022) 77:693–712. 10.1177/00207020231163063 37441228 PMC10333959

[36] Franke U Koch M. World sports and Russia’s war against Ukraine. In: Koch M, editor. Inter-Organizational Relations and World Order. Bristol: Bristol University Press (2023). p. 171–93.

[37] Kamenecka-Usova M Tkalych M. Resilience through sports: Ukraine and Latvia confront geopolitical challenges. Front Sports Act Living. (2025) 7:693–712. 10.3389/fspor.2025.1563761 PMC12183191 40552350

[38] Kuvaldina O Melo GLR Sarkauskiene A Taran L. Development and validation of a PEST-based instrument for assessing macro-environmental factors in Olympic athletes' preparation under crisis conditions. Front. Sports Act. Living (2026) 8:1856666. 10.3389/fspor.2026.1856666 42427914 PMC13345861

